# Measuring the Patients’ Satisfaction About Telemedicine Used in Saudi Arabia During COVID-19 Pandemic

**DOI:** 10.7759/cureus.13382

**Published:** 2021-02-16

**Authors:** Asmaa Abdel Nasser, Razan Mohammed Alzahrani, Chaimaa Aziz Fellah, Dimah Muwafak Jreash, Norah Talea A Almuwallad, Dunya Salem A Bakulka, Rabab Abdel Ra'oof Abed

**Affiliations:** 1 Medical Education Department, Faculty of Medicine, Suez Canal University, Ismailia, EGY; 2 Medical Education Unit, Ibn Sina National College for Medical Studies, Jeddah, SAU; 3 Medical Student, Ibn Sina National College for Medical Studies, Jeddah, SAU

**Keywords:** telemedicine, patients’ satisfaction, covid-19, telehealth

## Abstract

Background

Many studies have found that telemedicine and telehealth services quality and patients’ clinical outcomes, following telehealth visits, maybe comparable to those of traditional face-to-face office visits especially in a crisis like COVID-19 complete lockdown.

Objective

This study aimed to identify the patient's experience in using the telemedicine strategies during the COVID-19 pandemic and assess these patients' perception about their experience of using telemedicine in Saudi Arabia.

Methods

A cross-sectional survey study was done on 425 patients treated through telemedicine programs in Saudi Arabia from February to August 2020 during the COVID-19 pandemic in Saudi Arabia. An online questionnaire was adopted and modified to elicit participants’ socio-demographic data, participants’ satisfaction and attitude toward telehealth and telemedicine, and their views on health care services.

Results

About 84.9% of the participants thought that telemedicine made healthcare easier during the COVID-19 pandemic. Almost half of the respondent was very satisfied with the ease of registration (52%), while 43.4% of respondents stated that they had the ability to talk freely over telemedicine. In the present study, The highest satisfaction was reported by 53.4% of respondents for ease registration, 40.1% for quality of the visual image, 41.9% for quality of the audio sound, and 44.8% for their ability to talk freely over telemedicine, respectively. The highest satisfaction was reported by 40.5% about the ability to understand the recommendations, 40.5% about the overall quality of care provided, 37.4% about the overall telemedicine consult experience. The results revealed a significant positive correlation between satisfaction and attitude scores.

Conclusion

This study revealed acceptable satisfaction and attitude of patients toward telemedicine programs in Saudi Arabia. However, more effort should be done by the Saudi Ministry of Health to increase the knowledge of patients about teleconsultation available services.

## Introduction

In response to expanding technological developments in the world, healthcare systems require a paradigm shift in the way health services are delivered like telemedicine which supports using of electronic information and advanced telecommunication technologies to support long-distance clinical healthcare [1]. Telemedicine was defined by the World Health Organization as “the delivery of health care services by all health care professionals using technology for the exchange of valid information for the diagnosis, treatment, and prevention of disease and injuries” [2].

As coronavirus disease (COVID-19) is widely spreading across the Kingdom of Saudi Arabia (KSA) from the start of March 2020, patients have a fear that they will get infected when they go to hospitals to receive treatment, medical advice, and follow-up. Responding to that the patients expect to have diverse technologies that enable them to continue living in their house and be more informed and engaged in their own health and minimizing exposure to healthcare facilities [3].

Several studies have found that the quality of telehealth services and patients’ clinical outcomes following telehealth visits may be comparable to those of traditional face-to-face office visits, with the additional benefit of fast access to care [4], and a recent meta-analysis has found that teleconsultation provided a rapid alternative to face-to-face clinical visits [5]. It was also found to be an effective triage method to evaluate patients’ complaints, prevent unnecessary clinical visits, and reduce the waiting time [6].

During the pandemic, telemedicine was found effective in screening patients who may have symptoms of COVID-19. In addition, it was an appropriate method to provide low-risk urgent care for non-COVID-19 conditions, monitor clinical signs of certain chronic medical conditions, and follow up of patients after hospitalization [7]. A lot of countries implemented teleconsultation during the COVID-19 pandemic [8-11]. However, it is not currently known whether this available service has satisfied the patients' needs during the COVID-19 pandemic so for [12].

In June 2019, new regulations on telemedicine were published in the Kingdom of Saudi Arabia (KSA), providing a comprehensive framework for all clinical staff, which is overseen by the Saudi Telemedicine Unit of Excellence (STUE) as part of the National Health Information Centre. The publication of these regulations provides a foundation to rapidly implement video consultations across the Kingdom, hence, the use of telemedicine began to increase [13]. A recent study was done in Riyadh to measure satisfaction toward a tele-retinal screening program among diabetics attending endocrinology clinics at a tertiary hospital. The study found that patients were found to be highly satisfied with the tele-retinal screening program. At the same time, there is a reason for dissatisfaction which was the difficult accessibility to an ophthalmologist when a referral was needed [14].

According to a careful literature review, there is no published study from KSA assessing patients’ satisfaction with telemedicine during COVID-19. The aim of the present study was to assess the patients’ satisfaction with their experience of using telemedicine strategies under the COVID-19 pandemic.

## Materials and methods

Study type and setting

A cross-sectional survey study was done among patients who were treated from February to August 2020 through telemedicine programs all over Saudi Arabia.

Study participants and sampling methodology

The study was done on 425 patients who were treated through telemedicine programs in Saudi Arabia. Patients who were 18 years of age or older and who participated in telehealth visits or follow up (out-patient clinics in different specialties) during COVID-19 were included. The estimated online sample size by the equation for 50% prevalence of patients who used telemedicine during COVID-19 pandemics in Saudi Arabia revealed that our target sample should be 383 participants or more to have a confidence level of 95% that the real value is within ±5%. Participants were selected consequently using non-randomized voluntary response sampling [15].

Study instrument

Data were collected by an online questionnaire that was disseminated through various social media platforms to reach wide sectors of the Saudi community, which was adopted and modified to elicit participants’ socio-demographic data, participants’ satisfaction, and attitude toward telehealth and telemedicine, their views on healthcare services in Saudi Arabia during COVID-19 pandemic. The satisfaction of the participants was assessed by newly developed eight questions on a Likert scale that has five options. Each option was given a score that ranged from 1, which was given to the “strongly disagree” response, to 5 that was given to the “strongly agree” score. So, the satisfaction scores ranged from 8 to 40. The attitude of the participants towards telemedicine was detected by using five questions checklist with two responses; "yes” that was given a 2 score and “no” that was given a 1 score. So, the attitude score ranged from 5 to 10.

Ethical considerations

The Ethical clearance for the study was obtained from the Ibn Sina National College Research and Ethics committee (IEC Ref No.: H-24-19102020). All the participants were informed about the purpose of the study and their right to refuse participation. Ethical conduct was maintained during data collection and throughout the research process in accordance with the Helsinki Declaration [16]. Participation in the study was voluntary and the confidentiality of the participants was maintained as the questionnaire was provided anonymously. Each participant had the right to withdraw from the study at any point without any consequences.

Statistical analysis

Data analyzed by SPSS program version 23, where quantitative data was expressed as numbers and frequencies. Also, we used mean and standard deviation (mean ± SD) to measure the average and spread of participants’ responses and for non-parametric variables. Mann-Whitney, Kruskal Wallis tests, and Spearman’s correlation analysis were used. A p-value <0.05 was used as a cutoff point for statistical significance.

## Results

The collected responses were 425 who were treated through telemedicine programs in Saudi Arabia. Table 1 shows that 36.9% of the study participants had an age that ranged from 18 to 25 years, 63.1% were females, and 76.6% had a Saudi nationality.

**Table 1:** Distribution of the Studied Participants According to Their Characters and Being a Patient on the Telemedicine Network Before (n=425)

Variable		No (%)
Age	18–25	157 (36.9)
	26–35	85 (20)
	36–45	112 (24.5)
	45	71 (16.7)
Gender	Female	268 (63.1)
	Male	157 (36.9)
Nationality	Saudi	326 (76.6)
	Non-Saudi	99 (23.3)

Table 2 illustrates that most of the participants agreed that they think telehealth services made healthcare easier today during the COVID-19 pandemics. Furthermore, the majority of them thought that in case you need healthcare, they might have to miss work/get things done to see a therapist if telehealth services are not available. On the other hand, less than half of the participants will prefer telemedicine consultation in the future, and 80.2% of them thought that the presence of the camera and other equipment can embarrass them and make them feel uncomfortable. Table 3 shows that most of the participants responses ranged between very satisfied and satisfied especially for ease of registration/ scheduling (52%), quality of the visual image (38.8%), quality of audio, the ability to understand the recommendations or diagnosis made, and the comfort of telemedicine suite (40.5%), and ability to talk freely over telemedicine (43.5%)/37.4% and 36.7% were very satisfied with the overall quality of care provided, and the overall telemedicine consults experience, respectively.

**Table 2:** Distribution of the Studied Participants According to Their Response to Satisfaction Items Regarding Telemedicine (n=425)

Variable	Response				
	Very satisfied (%)	Satisfied (%)	Neutral (%)	Dissatisfied (%)	Very dissatisfied (%)
Ease of registration/scheduling	221 (52)	135 (31.8)	56 (13.2)	12 (2.8)	1 (0.2)
Quality of the visual image	165 (38.8)	167 (39.3)	73 (17.2)	18 (4.2)	2 (0.5)
Quality of the audio sound	172 (40.5)	160 (37.6)	78 (18.4)	12 (2.8)	3 (0.7)
Ability to talk freely over telemedicine	185 (43.5)	158 (37.2)	69 (16.2)	12 (2.8)	1 (0.2)
Ability to understand the recommendations or diagnosis made	72 (40.5)	169 (39.8)	61 (14.4)	21 (4.9)	2 (0.5)
The comfort of the telemedicine suite (the location where I received my care)	172 (40.5)	160 (37.6)	74 (17.4)	18 (4.2)	1 (0.2)
The overall quality of care provided	159 (37.4)	59 (37.4)	80 (18.8)	25 (5.9)	2 (0.5)
Overall telemedicine consult experience	156 (36.7)	175 (41.2)	79 (18.6)	14 (3.3)	1 (0.2)

**Table 3:** The Participants’ Attitude Towards Telemedicine (n=425)

Variable		No (%)
Do you think telehealth services made healthcare easier today during the virus COVID-19 pandemic?	Agree	361 (84.9)
	I do not agree	64 (15.1)
In case you need healthcare, do you think you might have to miss work/get things done to see a therapist if telehealth services are not available?	Agree	344 (80.9)
	I do not agree	81 (19.1)
In the future, which would you prefer?	Telemedicine consultation	208 (48.9)
	Face-to-face consultation	217 (51.1)
Would you be willing to participate in another telemedicine consultation?	Yes	171 (40.2)
	No or not sure	254 (59.8)
Do you think the presence of the camera and other equipment can embarrass you or make you feel uncomfortable?	Agree	341 (80.2)
	I do not agree	84 (19.8)

**Figure 1:**
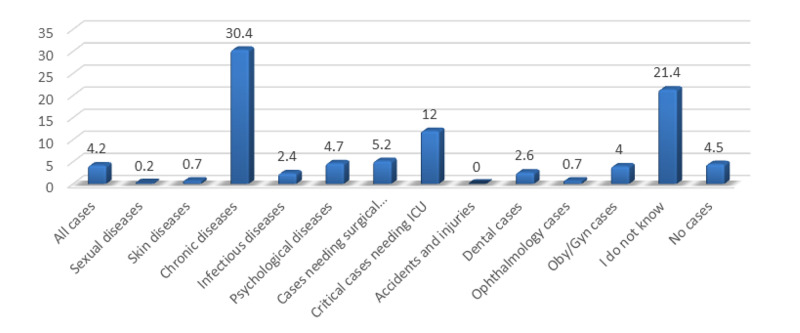
Distribution of the Studied Participants’ Opinion About the Most Suitable Diseases to be Managed by Telemedicine (n=425)

The participants reported that if telemedicine had not been available for their consult today, they would drive to meet the specialist face-to-face (43.5%), and 34.1% reported that they would travel for less than 15 minutes to receive care. The participants reported that if telemedicine had not been available and they had to travel to meet face-to-face with the provider, the most affected item would be that their companions would have lost time from work (35.5%). Only 4.2% of the participants thought that telemedicine is suitable for all medical cases, and they thought that chronic diseases are the most suitable to be cared for by telemedicine (30.4%; Table 4 and Figure 1).

**Table 4:** Frequency of the Participants’ Responses to Different Items Related to Telemedicine (n=425)

Items		No (%)
If telemedicine had not been available for your consult today, which of the following would have been your alternative plan of action?	I would have driven to see the specialist face-to-face	185 (43.5)
	I would have contacted my local clinical to see if they could assist	100 (23.5)
	I would not go see any doctor	32 (7.5)
	The use of alternative medicine (honey - nigella - Indian installment, etc.) 12,111	108 (25.4)
If telemedicine had not been available for your consult today, how far would you have had to travel to receive care?	Less than 15 minutes	145 (34.1)
	15-30 minutes	139 (32.7)
	30 minutes to 1 hour	78 (18.4)
	1-2 hours	49 (11.5)
	More than 2 hours	14 (3.3)
If telemedicine had not been available and you had to travel to meet face-to-face with the provider to receive care, which of the following would apply?	I would have lost time from work	114 (26.8)
	My companions would have lost time from work	151 (35.5)
	I would have paid for meals while I was away from home	55 (12.9)
	I would have paid for a hotel to spend the night	105 (24.7)
Do you think telemedicine is suitable for all medical cases?	No	269 (63.3)
	Yes	156 (36.7)

The mean satisfaction and attitude scores were 33.24 ± 5.94 and 9.72 ± 1.24, respectively. Participants with an age ranging from 18 to 25 years and those of Saudi nationality had a significantly higher mean satisfaction score compared to other participants (p≤0.05). While a non-significant difference was found between satisfaction scores and participants' gender and being a patient on the telemedicine network before (p≥0.05; Table 5).

**Table 5:** Relationship Between Participants’ Mean Satisfaction Score and Their Characters (n=425) *Statistically significant p-value < 0.05. **Kruskal-Wallis test. ***Mann-Whitney test.

Variable		Satisfaction score (mean ± SD)	Test	p-Value
Age	18–25	34.4 ± 5.74	3**	0.002*
	26–35	32.69 ± 6.31		
	36–45	33.12 ± 5.91		
	>45	31.52 ± 5.56		
Gender	Female	33.5 ± 6.33	1.18***	0.23
	Male	33.08 ± 5.71		
Nationality	Saudi	33.68 ± 5.77	2.64***	0.008*
	Non-Saudi	31.8 3 6.29		

As for participants' attitudes towards telemedicine, female participants had a significantly higher mean attitude score compared to other participants (p≤0.05). On the other hand, a non-significant difference was found between satisfaction scores and participants’ age and nationality (p≥0.05; Table 6).

**Table 6:** Relationship Between Participants’ Mean Attitude Score and Their Characters (n=425) *Statistically significant p-value <0.05. **Kruskal-Wallis test. ***Mann-Whitney test.

Variable		Attitude score (mean ± SD)	Test	p-Value
Age	18–25	8.2 ± 1.01	3**	0.17
	26–35	8.4 ± 1.04		
	36–45	8.58 ± 1.02		
	>45	8.19 ± 1.09		
Gender	Female	8.4 ± 1.01	3.03***	0.002*
	Male	8.37 ± 1.06		
Nationality	Saudi	8.35 ± 1.03	0.64***	0.52
	Non-Saudi	8.33 ± 1.09		

**Figure 2:**
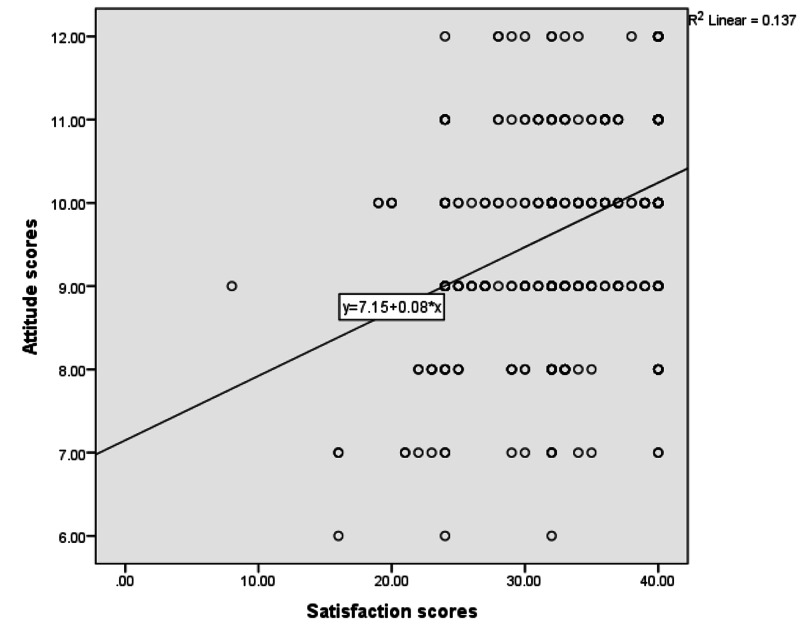
Spearman’s Correlation Analysis Between Satisfaction and Attitude Scores (n=425) N.B.: (r=0.35, p≤0.001).

Figure 2 demonstrated a significant positive correlation between satisfaction scores and attitude scores (r=0.35, p≤0.001).

## Discussion

This research work aims to measure the patients' satisfaction regarding the use of tele-healthcare services during the COVID-19 pandemic in KSA. Traditionally, healthcare encounters a relationship between a provider and a patient being in the same place (in person) [16]. Over the past 20 years, the internet and technology have made it possible for healthcare to be delivered digitally, providing new avenues for the medicine to improve the value of care [17]. The strengths of telemedicine have made it an indispensable tool in the clinical response to the COVID-19 pandemic [18]. With the removal of financial disincentives and privacy barriers that limited widespread adoption of telemedicine, the Saudi Vision 2030 framework, released in 2017, has paved the path for technology transformation, the pandemic of COVID-19 enabled the promotion and testing of this transition [19].

Most of the participants realize that telehealth services made healthcare easier today during pandemics or any similar situation. Most of them were very satisfied with the ease of registration/scheduling, quality of the visual image, quality of the audio sound, and ability to talk freely over telemedicine. In this context, telemedicine consultation got around the restrictions of lockdown, making follow-up possible; this may well account for the high rate of satisfaction, but also means the results cannot be extrapolated to telemedicine in general [20].

Prior studies have shown similar high patients’ satisfaction with telemedicine (4), and clinical outcomes of patients using telemedicine services were found to be comparable with those using traditional “in-person” clinic visits [21,22]. However, these studies were done in the developed countries where telemedicine is better established than it is in KSA and many other countries around the world [23].

In this study, most of the participants reported that if telemedicine had not been available, and they had to travel to meet with their health care provider face-to-face, their companions would have lost time from work [20]. The participants thought that telemedicine is not suitable for all medical cases, and most of them thought that chronic disease follow-up is the most suitable to be cared for by telemedicine. A systematic review done in 2019 supported our result and showed that to manage COVID-19, there are many easy-to- set-up potentials in live video consulting [24]. Studies have found that live video conferencing can lead to the avoiding of direct physical contact, thereby diminishing the risk of exposure to respiratory secretions, and preventing the potential transmission of infection to physicians and other healthcare providers [25]. Also, the live video could be very useful for patients seeking consultation on COVID-19, for people with heightened anxiety, instead of in-person visits in cases of chronic disease reviews (such as diabetes and cancer), some medication checks, and triage when the telephone is insufficient [26].

In this work, the participants were very satisfied with the ability to understand the recommendations or diagnosis made, the comfort of the telemedicine suite, the overall quality of care provided, and the overall telemedicine consult experience, respectively. It was found that to control the COVID-19 outbreak the spread, video consultations, and telephone follow-up is possible in multiple cancer settings [27]. A study done in the USA showed that electronic health records (EHR) and phone calls facilitated screening or treating a patient without the need for in-person visits and improve the decision-making process among healthcare workers in ambulatory and urgent care [28].

Most of the old age participants in our study would prefer to use face-to-face consultation in the future rather than telemedicine; this may be due to their age generation and they need in-person contact to discuss more their emotional issues, and to express all his/her concerns verbally and nonverbally. There was a significant positive correlation between patients’ satisfaction and attitude scores toward telemedicine. Many studies highlighted the impact of telehealthcare services during the COVID-19 pandemic for keeping social distancing from high-risk areas, which will prevent morbidity [24].

As for participants’ satisfaction and attitude towards telemedicine, participants with an age ranging from 18 to 25 years had a significantly higher mean satisfaction score compared to other participants, this may be for the reason that they are young age with more familiarity with the use of technology. A non-significant difference was found between satisfaction scores and the participants’ gender, as the female participants had a significantly higher mean attitude score compared to other participants. Also, a non-significant difference was found between satisfaction scores and participants' age and nationality. In a previous study, predictors of liking telehealth were female gender, being very satisfied with and understanding of telehealth, and quality of care received [4].

The barriers encountered to adopt telehealth services for large-scale use during COVID-19 infection are the adaptation of health systems with rapid changes regarding payment and coordination of services [29]. Despite showing an overall high satisfaction rate with the telemedicine services in this study, we should take into our consideration the possible response bias that can affect the outcome. In addition, the results of this study cannot be generalized to all medical subspecialties as the applicability of telemedicine consultations in different subspecialties must be determined individually.

## Conclusions

The COVID-19 pandemic is making changes and increasing the need to apply telehealth more firmly. The current study revealed adequate satisfaction and attitude of patients towards telemedicine consultation services at the lock down time of COVID-19. However, more effort should be done by the Saudi Ministry of Health (MOH) to increase the patients' awareness and knowledge about teleconsultation services. Future studies assessing physicians’ perception toward Telemedicine and teleconsultation options should be encouraged.

## Appendices

If you participated in telehealth consultation services during the COVID-19 pandemic, please specify do you would like to participate in this research?

I want

I do not want

1. Age:

18- 25

26 -35

36 - 45

46 - 55

2. Sex:

Man

Woman

3. What is Your Nationality?

Saudi

Non-Saudi

4. Is the first time you have been seen as a patient on the telemedicine network?

Yes

No

5. Mention the hospital (center) where you have an appointment for telemedicine?

6. What physician and location were you connecting with?

7. How would you rate your telemedicine consultation on the factors listed below?

**Table 7:** Factors for Telemedicine Consultation

	Very satisfied	Satisfied	Neutral	Dissatisfied	Very dissatisfied
Ease of registration/scheduling					
Quality of the visual image					
Quality of the audio sound					
Ability to talk freely over telemedicine					
Ability to understand the recommendations or diagnosis made					
The comfort of the telemedicine suite (the location where I received my care)					
The overall quality of care provided					
Overall telemedicine consult experience					

8. Do you think telehealth services made healthcare easier today during the virus Covid 19 pandemic?

Agree

I don't agree

9. In case you need healthcare, do you think you might have to miss work/get things done to see a therapist if telehealth services are not available?

Agree

I don't agree

10. If telemedicine had not been available for your consult today, which of the following would have been your alternative plan of action?

I would have driven to see the specialist face-to-face

I would have contacted my local clinical to see if they could assist

I wouldn't go see any doctor 

The use of alternative medicine (honey - nigella - Indian installment, etc.)

Other ……

11. If telemedicine had not been available for your consult today, how far would you have had to travel to receive care?

Less than 15 minutes

15 - 30 minutes

30 minutes - 1 hour

1-2 hours

More than 2 hours

Other (please specify)

12. *If telemedicine had not been available and you had to travel to meet face-to-face with the provider to receive care, which of the following would apply? (please check all that apply)

I would have lost time from work

My companions would have lost time from work

I would have paid for meals while I was away from home

I would have paid for a hotel to spend the night

Other expenses (please specify)

13. In the future, which would you prefer?

Telemedicine Consultation

Face-to-face Consultation

14. Would you be willing to participate in another telemedicine consultation?

Yes

No

Not sure

15. Do you have any suggestions for improving consultations?

Mention it.......

16. What are the cases or diseases that are not suitable for telemedicine?

Mention it.......

17. Do you think the presence of the camera and other equipment can embarrass you or make you feel uncomfortable?

Agree

I don't agree
